# VCNet: Optimized Deep Learning framework with deep feature extraction and genetic algorithm for multiclass rice crop disease detection

**DOI:** 10.1016/j.mex.2025.103551

**Published:** 2025-08-05

**Authors:** Sanam Salman Kazi, Bhakti Palkar, Dhirendra Mishra

**Affiliations:** a Department of Computer Engineering, K. J. Somaiya School of Engineering (KJSSE), Somaiya Vidyavihar University, Mumbai, Maharashtra 400077, India; b Department of Engineering and Technology, Bharati Vidyapeeth Deemed to be University, Kharghar, Navi Mumbai, Maharashtra 410210, India; c Department of Computer Engineering, SVKM’s NMIMS Deemed to be University Mukesh Patel School of Technology Management & Engineering, Mumbai, 400056, India

**Keywords:** Deep learning, Rice disease detection, Optimization, Multiclass Classification, Transfer learning

## Abstract

Convolution Neural Networks (CNN) are best in their ability to detect rice diseases but still face challenges in generalizing equally well for all classes of disease in multiclass classification. Detecting rice crop disease like sheath rot is still challenging due to unavailability of dataset and intraclass variations in symptoms. Transfer learning models take more resources for execution due to its deep architecture. To conquer these challenges, VCNet, an optimized, novel and efficient multiclass rice crop disease detection framework is proposed. The study focuses on developing a shallow model with deep feature extraction to bring down the computational load with reduced time for training without compromising on any performance parameters. Further the model goes through two level optimization process where optimal hyperparameters identified through experimentation is given as parameters to genetic algorithm for optimization of VCNet during training. Novel dataset containing field images is generated with the help of plant pathologist to improve model capability to identify diseases. Rigorous empirical comparison and evaluation with state-of-the-art models for each class of disease is done to validate proposed technique. VCNet outperforms the existing transfer learning models with training accuracy 99.72 % and testing accuracy 97.71 %. It also requires fewer parameters and takes minimum training time.

• The major contribution of this study is the design of an optimized, efficient and enhanced deep learning technique for multiclass rice crop disease detection embracing with batch normalization, dropout and genetic optimization algorithm to improve generalization power and restrict the overlearning capability for seen and unseen data.
• Proposed VCNet, a shallow model with deep feature extraction, employs VGG16 layers for initial extraction fused with custom CNN architecture to correctly detect the challenging classes of diseases like sheath rot in multiclass classification.
• The most significant observation is that VCNet accurately predicts the rice disease for each class of diseases under study whereas the existing powerful models largely misclassified for some classes of diseases in multiclass classification.

## Graphic abstract

**Figure:**
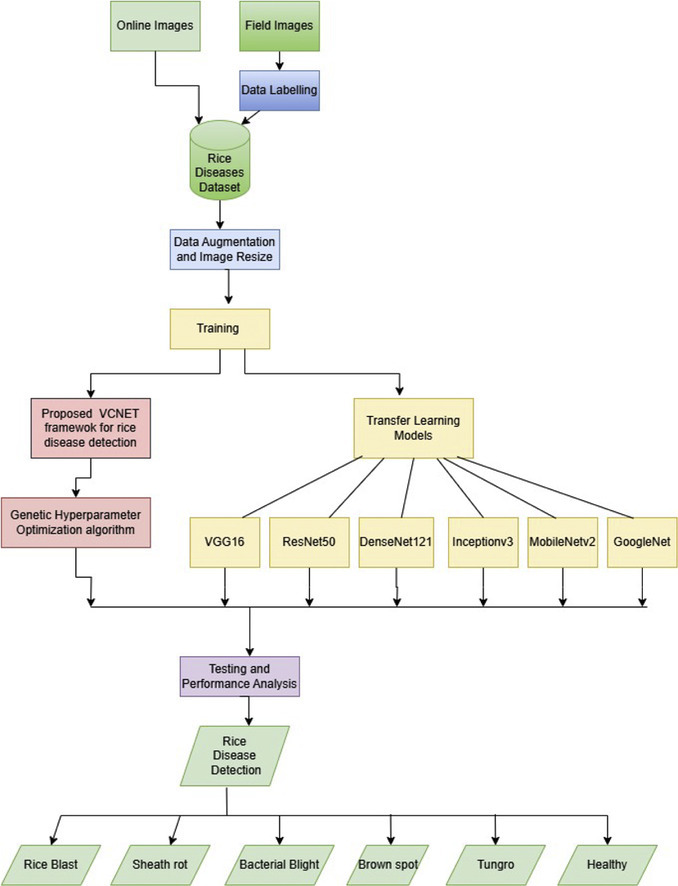

Specifications table

**Table:** 

**Subject area**	Computer Science
**More specific subject area**	*Artificial Intelligence, Deep Learning*
**Name of your method**	*VCNet*
**Name and reference of original method**	*NA*
**Resource availability**	*NA*

## Background

Rice cultivation faces serious challenges brought about by various diseases to seriously affect crop yield and quality. Among them, rice blast, bacterial blight, sheath rot, tungro, and brown spot are the more harmful ones that cause huge losses every year all over the world. The conventional methodology for their detection and management involves the gradual manual observation of the disease by field experts. It can lead to unreliable outcome. According to Bock, Chiang, and Del Ponte, despite research into more efficient methods of plant disease severity assessment by visual estimation, there is still a strong demand for the development of more effective approaches due to the problems with consistency and accuracy that still persist [1]. CNNs automate the feature extraction process, where thousands of images are analyzed and it learns to identify intricate patterns associated with different diseases. This capability boost accuracy and speeds up the process enabling real-time monitoring and diagnosis across large areas. Notable models such as VGG16, DenseNet121, InceptionV3, GoogleNet, MobileNetV2, and ResNet50 have been adapted for agricultural applications, demonstrating high efficacy in recognizing crop diseases. Andrew et al. (2022) [2] studied processing of agricultural image data for feature extraction by leveraging the robustness of a CNN. Kumar et al. (2023) [3] used a YOLO with a bidirectional feature attention pyramid network to demonstrate improved detection accuracy and speed arising from the integration of a complex architecture. On the other hand, the major improvement in disease detection comes with transfer learning. As the name goes it means that a model should be pre-trained on a large dataset, normally a general image recognition dataset, and fine-tuned on some small dataset, containing images of the target application. This method has been effective since it allows the models to learn from a vast amount of visual data and then apply the learned knowledge to the specific task of plant disease identification, which might have limited examples for training. This idea is materialized in Gopi et al. [4], where the transfer learning approach reduces the number of large volumes of labeled datasets needed for each kind of disease, which are usually cumbersome and costly to compile.

The major contribution in the proposed research paper is the design of a novel optimized and enhanced rice disease detection framework to improve generalization power of each class in multiclass classification. The focus of this study is be the optimization of proposed model in an effort to achieve a trade-off between model accuracy, efficiency in training, and complexity by using VGG16 for preliminary feature extraction and further enhancing it with custom CNN layers. The shallow model using deep feature extraction with VGG16 is employed with Genetic algorithm for optimization. Two step optimization process for hyperparameters during training plays vital role in enhancing model performance. An enhanced and optimized model would then be drawn from a rigorous comparison with state-of-the-art architectures, like MobileNetV2, DenseNet121, Inception v2, GoogLeNet, and ResNet50 [5]. The dataset is novel and balanced with field collected images. To increase generalization capability the images are preprocessed and augmented.

## Method details

**Fig. 1:**
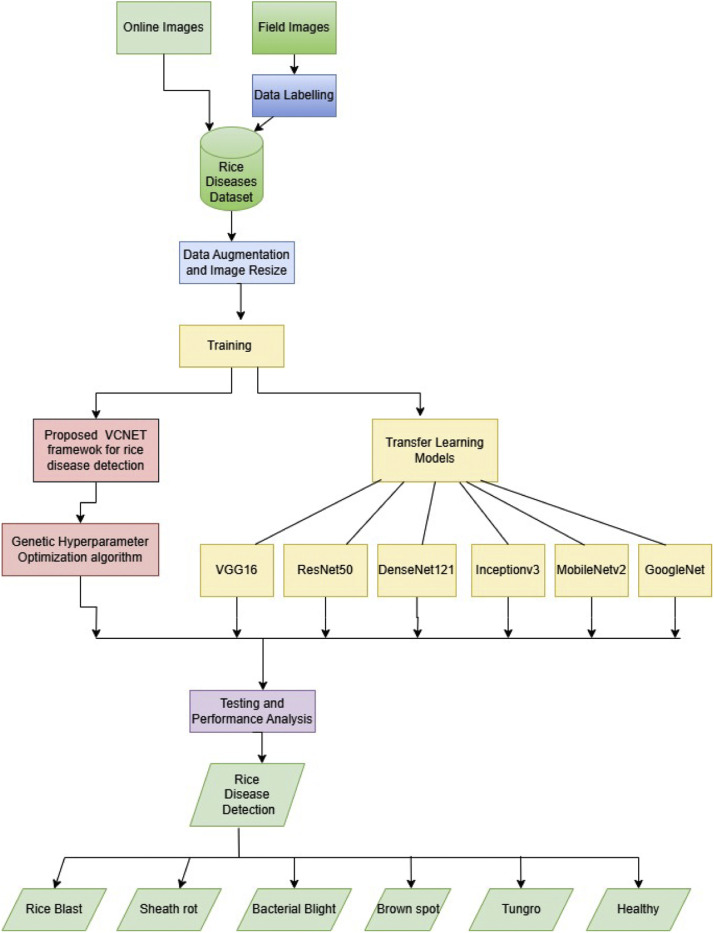
Comprehensive workflow for proposed methodology.

Improvement in rice disease classification would, therefore, follow the methodology involving a CNN architecture, leveraging VGG16 model initialization for initial feature extraction, followed by adding custom CNN layers that better recognize diseases. This will involve the tuning of important hyperparameters by employing Genetic algorithm to optimize the learning rate, number of neurons, dropout rate for optimum performance both at accuracy and training efficiency. The enhanced model focuses on bringing down the computational load with reduced time for training by not compromising on any performance parameters. The proposed VCNet model further compares other leading architectures in the field concerning MobileNetV2, DenseNet121, Inceptionv3, VGG, and ResNet50 to validate and set a benchmark for its effectiveness. These comparatives are made with accuracy and performance so that not only the current benchmark standards are met but a step ahead. The Fig. 1 below highlights the summarized workflow for experimenting the proposed methodology.

Various deep learning technologies have significantly improved plant disease detection in terms of accuracy, efficiency, and adaptability for different agriculture-related scenarios. Recent contributions have underlined the very effectiveness of such technologies for rice crops. Starting with the improvements in CNNs, Bari et al. (2021) [6] attained accuracy 99.25% using the R-CNN for categorizing four rice diseases. This model was privileged with a hybrid dataset of self- generated collection and images from Kaggle, totaling 2400 images, showing strengths for integrated data sources and advanced computational frameworks. As an extension of the developments at CNN, Pandian et al. (2022) [7] used evolutionary search to optimize a ResNet19 based model using different augmentation methods on the dataset. The resultant model, ResNet197 had outstanding classification accuracy of 99.58 %, showcasing superiority against traditional architectures and demonstrated an opportunity for deep learning mechanisms over complex image recognition tasks within agriculture. Following Table 1 summarizes the methods adopted by researchers and its impact analysis.

**Table 1:** Analysis of existing methodologies for rice disease detection.

Paper	Methods	Dataset	Robustness	Computational Efficiency	Classification Accuracy ( %)
[8], 2021	Random Forest	Self-generated rice leaf database	High	Moderate	99.2
[9], 2023	ResNet 50	International Rice Research Institute (IRRI)	High	High	99.2
[10], 2022	Thepade SBTC and Otsu thresholding	Self-generated database	Moderate	Low	85.9
[11], 2020	CNN	Self-generated	High	Moderate	97.35
[12], 2023	Hybrid CNN-Efficient Net B7	Plant Village dataset	Moderate	Low	88.93
[13], 2022	SVM with spectra pre-processing	Self-generated	High	High	98.7
[14], 2022	ResNet34, ResNet 50, ResNet 18	Public dataset	Moderate	Moderate	93
[15], 2023	DenseNet 169, Xception	Public dataset	High	Low	98.54
[16], 2023	AlexNet, VGG16, CNN	Benchmark datasets	High	Moderate	98.05
[17], 2023	ResNet 50	Benchmark dataset	High	High	99.66, 99.9

Hybrid models, on the other hand, were developed by Kaur et al. (2022) [13], who developed a hybrid convolutional neural network that applied feature reduction to reduce processing load, a key factor for deployment on edge devices. Although this did indeed reduce computational burden, there was a risk of narrowing the generalization capacity for disease detection. Saberi Anari (2022) [18] introduced an ME learning approach for Model Engineering. Deep transfer learning was integrated with several models of SVM to improve the classification of diseases in leaves. These included k-NN, DT, NN, SVM-L, SVM-RBF, and ensemble models, all combined for better recognition capability. More recent studies further develop the precision and application specificity of such models. Zhou et al. (2023) [19] and Velusamy et al. (2023) [20] proposed improved disease detection accuracy with better CNN architecture and hybrid models, achieving classification accuracies as high as 98.26 %. These works not only improve disease identification, but also adapt to changes in agricultural conditions, as represented by Haridasan et al. (2023) [12], who specifically addressed challenges in the disease of paddy and rice leaves.

Deng et al. (2021) [21] studied an extended number of rice leaf diseases; employing a Faster-RCNN model, pre-trained on as many as 33,026 images, with a detailed review of methodologies and dataset characteristics. This extensive approach underlines the scalability and detailed analytical capabilities of modern deep learning models. Javidan et al. (2023) [22], and other researchers have adopted an integrated approach, combining machine learning techniques such as K-means clustering with deep learning frameworks to further improve disease detection. However, these models sometimes cannot capture all the nuances associated with plant pathology, which may be critical for identifying complex disease presentations. Shoaib et al. (2023) [23] give a broad perspective:” Future improvements are suggested to be done by improving datasets and model training methodologies that will lead to high performance universally across different crops and disease types.” This is, therefore, an important perspective to keep in mind for further work in the area of developing more robust and efficient, widely applicable systems for plant disease detection.

The economic benefits of improving plant disease detection are also considerable. Enhanced accuracy and early detection capabilities can drastically reduce crop losses, boosting profitability and stabilizing food markets, particularly in regions heavily reliant on agriculture. However, efficiency and accuracy in the deep learning models for field images is still a challenge and is restricted by the general unavailability of quality data with proper annotation, something very much essential for its training. Expanding it to multiclass classification for multiple diseases would bring a big paradigm shift in model performance as well as adaptability. These factors, therefore, set a very strong case for continued research and development into AI- driven plant disease detection, which promises not only the enhancement of crop disease detection capability but also significant contributions toward ensuring efficiency of the models.

## Data collection

The dataset used for this study consists of 9346 RGB images, resized to 150 × 150 pixels, from two major sources: Dapoli farms and Karjat research farm, supplemented by images publicly available on platforms like Kaggle and Mendeley. This collection is distributed across six categories reflecting different states of rice plant health, including several disease conditions and healthy specimens. It consists of 1737 rice blast images, 1994 of bacterial blight, 1220 of sheath rot, 1307 of tungro, 1600 of brown spot, and 1488 images of healthy rice plants. 7467 images for training and 1879 reserved for testing. This will lay a strong structure in training the model to identify and classify various rice diseases, which is important for disease management and increasing agricultural productivity. The images are selected such that dataset possess symptoms and background variability to make the training reliable and robust.

## Data labelling and augmentation

**Fig. 2:**
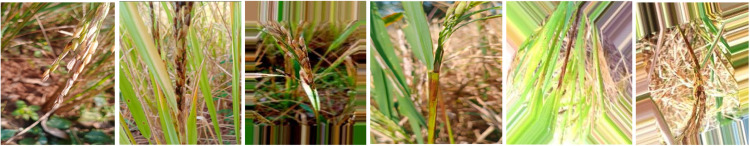
Dataset with symptoms variability and augmentation for Sheathrot disease.

Data labelling and augmentation are the most critical and initial steps in the process of training CNN models. First, the images collected are manually labelled with the help of plant pathologist from Agricultural university at as rice blast, sheath rot, bacterial blight, brown spot, tungro, and healthy plants. This labelling is quite important because it sets the ground truth necessary for efficient supervised learning. This is followed by several data augmentation techniques, which improve the generalization capability of the model. These include rotations, scaling, and color adjustments that help in simulating different variations of the same disease under different conditions. Following Fig. 2 illustrates the same. The images are then resized to a dimension of 150 × 150 pixels. This standardization allows the sizes fed into the respective neural networks to be uniform when receiving incoming data; thereby, it enhances the said processing to be more sufficient or accurate during the training session.

## Proposed VCNet framework

VGG16 serves as an essential component of this framework for deep feature extraction. The VGG16 consists of 5 convolutional layer blocks that increase in depth one after another, therefore extracting complex features at different scales. This pre-trained VGG16 model gives the network a vast number of learned visual features, therefore greatly enriching its generalization capability when dealing with different and unseen rice disease images. The VGG16 architecture is adapted for rice disease detection by tuning the model on specific characteristics of rice pathology images. This involves fine-tuning the top layers of the VGG16 model to better align with the specifics of the rice disease dataset. The fully connected layers of the VGG16 are replaced with new layers that are trained from scratch to classify rice diseases into multiple classes. This ensures that the network learns features that are more relevant to the specific types of disease that affect rice crops than to the more general features learned from ImageNet. Besides adapting VGG16, further custom CNN layers are added on top to make the model more predictive. The idea is to let these layers work in conjunction with the deep features extracted from VGG16 for a more fine-grained analysis of the input images. This involves adding convolutional layers, dropout layers to avoid overfitting, and dense layers to do the final classification. Each of these layers adds up to the strength in the detection mechanism, enabling it to bear the variability and complexity of field images of rice crops.

**Fig. 3:**
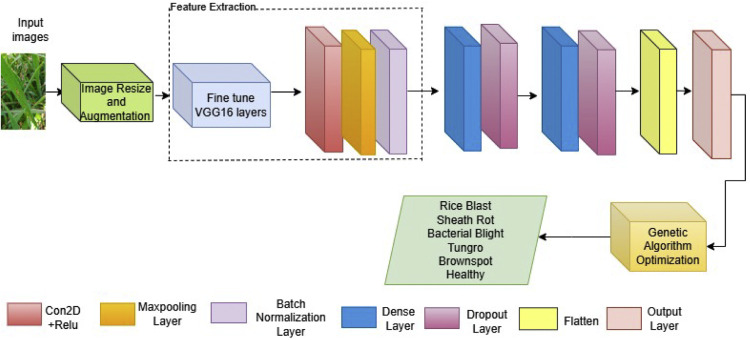
Proposed VCNet framework for multiclass rice disease detection.

This Fig. 3 illustrates a deep architecture adopted for the classification of different plant diseases of rice using the convolutional neural network-VGG16 architecture model. The entire process undergoes different stages, starting with placing the rice leaf images on the input. Pre-processing includes converting every input to 150 * 150 pixels followed by data augmentation to increase the size and generalize the model [24]. Initial feature extraction is done using the VGG16 model, which is one of the most efficient for capturing complex features in image data. The convolutional layers in VGG16, apply numerous filters to the input images, transforming them through nonlinear ReLU(Rectified Linear Unit) activations defined by the equation:

(1) f(x)=max(0,x)

where the output for given positive input x is x and for negative input the output is 0. Following convolution, the architecture employs max pooling with a 2 *2 window, down sampling the spatial dimensions (width and height) of the convolved features to shrink the image representation and reduce computational load. The max pooling operation for feature map X is expressed as:

(2) $$Y_{i,j}=\underset{\left(m,n\right)\in R_{i,j}}{\text{max}}X_{m,n}$$

where $R_{i,j}$ is the area in the input for pooling window at location (i,j). Max pooling reduces redundancy by maintaining only maximum value. After pooling, batch normalization normalizes the activations from the prior layer, enhancing the stability and speed of the network’s training phase. The transformation function for batch normalization is given as:

(3) $$x_{i}=\gamma \cdot \frac{x_{i}-\mu_{B}}{\sqrt{\sigma_{B}^{2}+\varepsilon }}+\beta \;$$

where $\mu_{B}$ and $\sigma_{B}^{2}$ are the mean and variance of the batch, ε is a small number to avoid division by zero, and γ andβ are parameters to be learned.

The VGG16 feature extractor contains five blocks containing convolutional layers and Maxpooling as shown in Table 2. The image x of size 4 × 4 and extracted features from block 5 of VGG16 are given further to custom CNN block 6 where the final features are extracted using added convolutional layer to make the model learn deep essential features to improve generalization on unseen data. It is then passed through max pooling and batch normalization layers to remove redundant features. The features are then fed into two dense layers, fully connected layers, with dropout applied in between to prevent overfitting. Dropout randomly sets a fraction of input units to 0 at each update during training time, which helps in preventing co-adaptation where neurons fail to learn independently. The dropout rate after the first dense layer is 0.4, and before the final output layer is 0.5. The dense layer with increasing neurons (125,150) and dropout (0.5, 0.4) is key design choice to obtain accurate prediction in output layer with six classes. The Table 2 below represents the mathematical process for the Fig. 3.

**Table 2:** Deep feature extraction methodology for proposed VCNet framework.

Initial Feature Extraction using fine tuned VGG16 Layers	Deep Feature Extraction using Custom CNN layers	Mathematical Process
block1: Conv2D(Filters:64, image size: 150×150, kernel: 3 × 3)	block6: Conv2D(Filters:128, image size:4 × 4, kernel:3 × 3)	$y_{1}=\varphi \left(Con2D\;\left(x;W_{1}\right)+b_{1}\right)$
block1: Conv2D(Filters:64, image size: 150×150, kernel:3 × 3)	block6: Maxpooling2D (2 × 2, image size:2 × 2)	$y_{2}=Maxpool2D\left(y_{1}\right)$
block1: Maxpooling2D(2 × 2, image size: 75×75)	block6: Batch Normalization Layer	$y_{3}=BatchNormalization\left(y_{2}\right)$
block2: Conv2D(Filters:128, image Size: 75×75, kernel:3 × 3)	DenseLayer(Neurons:125, activation:ReLU)	$\;y_{4}=\varphi \left(Dense(y_{3};W2)+b2\right)$
block2: Conv2D(Filters:128, image size: 75×75, kernel:3 × 3)	DropoutLayer(0.4)	$y_{5}=\text{Drop}out\left(y_{4},0.4\right)$
block2: Maxpooling2D(2 × 2, image size: 37×37)	DenseLayer(Neurons:150, activation:ReLU)	$y_{6}=\varphi (Dense\left(y_{5};W3)+b3\right)$
block3: Conv2D(Filters:256, image size: 37×37, kernel:3 × 3)	DropoutLayer(0.5)	$y_{7}=Dropout\left(y_{6},0.5\right)$
block3: Conv2D(Filters:256, image size: 37×37, kernel:3 × 3)	Flatten	$y_{8}=Flatten\left(y_{7}\right)$
block3: Conv2D(Filters:256, image size: 37×37, kernel:3 × 3)	DenseLayer(Neurons:6, activation:Softmax)	$Pred=Softmax(Dense(y_{8};W4)+b4)$
block3: Maxpooling2D(2 × 2, image size: 18×18)		
block4: Conv2D(Filters:512, image size: 18×18, kernel:3 × 3)		
block4: Conv2D(Filters:512, image size: 18×18, kernel:3 × 3)		
block4: Conv2D(Filters:512, image size: 18×18, kernel:3 × 3)		
block4: Maxpooling2D(2 × 2, image size: 9 × 9)		
block5: Conv2D(Filters:512, image size: 9 × 9, Kernel:3 × 3)		
block5:Conv2D(Filters:512, Image size: 9 × 9, kernel:3 × 3)		
block5:Conv2D(Filters:512, Image size: 9 × 9, Kernel:3 × 3)		
block5:Maxpooling2D (2 × 2, image size: 4 × 4)		

Lastly, the network flattens the output of the final pooling layer into a long feature vector, which is processed by the dense layers for classification. The output layer includes units equal to the number of classes, each using the softmax function to compute probabilities across all classes, which include rice blast, sheath rot, bacterial blight, tungro, brown spot, and healthy leaves. The softmax function for a class i out of k total classes is given by:

(4) $$z_{i}=\frac{e^{z_{i}}}{\sum_{j=1}^{k}e^{z_{j}}}\;$$

Where $z_{i}$ is the logit. This function converts the output layer’s logits into probabilities by exponentiating and normalizing each output, thus providing a probabilistic interpretation suitable for multi-class classification. The Adam optimization algorithm uses adaptive learning rate mechanism, which calculates individual learning rates for different parameters.

The process of optimization uses technique genetic algorithm in order to find the best set of parameters that yield the highest classification accuracy on validation datasets. The training process involves exposing the model to a huge dataset of labelled images of rice diseases, where it is able to learn the discrimination of healthy versus infected rice plants. The validation phase tests the effectiveness of the model on different sets of images that ensure it generalizes well to new data. This phase is very important for fine-tuning the model parameters and for making adjustments to either the model architecture or the training process to enhance performance. Indeed, the proposed deep learning framework, which considers deep features with the VGG16 model tuned to certain parameters, combined with the integration of extra CNN layers, contributes much toward a high identification performance of rice diseases. The development contributes not only to timely and precise control but also to an improvement in agricultural productivity as a whole (Algorithm 1, Algorithm 2).

**Algorithm 1:** Advanced deep learning framework for multiclass rice disease detection-VCNet.

Result: Optimized deep learning model for accurate and efficient multiclass classification of rice diseases
Initialization:
Load VGG16 pre-trained on ImageNet, excluding the fully-connected top layers, to leverage learned
hierarchical feature representations;
Append custom convolutional layers: Integrate additional one Conv2D and MaxPooling2D layers to extend feature extraction capabilities specific to rice disease patterns;
Attach a fully connected network: Configure multiple Dense layers tailored for classifying the specific classes of rice diseases;
Incorporate regularization mechanisms: Implement dropout layers strategically to mitigate overfitting during training;
Initialize model hyperparameters: Set initial parameters to optimal value obtained through experimentation and allow provisions for dynamic adjustments during training using Genetic algorithm;
Training procedure:
for each epoch do
for each minibatch in the training dataset do
Execute a forward propagation pass using current model parameters;
Compute the loss function (categorical cross-entropy) to gauge discrepancies between predicted and actual labels;
Apply backpropagation: Calculate gradients and update model parameters using an Adam optimizer;
end
Conduct validation: Periodically assess model performance on a held-out validation set to prevent overfitting and monitor generalization ability;
Dynamically adjust parameters to enhance convergence;
end
Testing and Performance Evaluation:
Load independent testing dataset;
Perform inference using the fine-tuned model to classify new data;
Compute key performance indicators: Accuracy, Precision, Recall, and F1-Score, alongside generating a confusion matrix for detailed error analysis;
Hyperparameter optimization and model refinement:
if validation performance metrics do not meet predefined thresholds, then
Employ hyperparameter tuning genetic algorithm, to find optimal model settings;
Experiment with adding or removing layers, adjusting layer sizes, or modifying dropout rates; Reiterate the training process with refined parameters to achieve improved model performance;
end

**Algorithm 2:** Genetic algorithm for optimizing model with hyperparameters.

Input: Number of generations, population size, fitness threshold
Output: Optimized model hyperparameters
Function Main:
Population ← InitializePopulation(population size);
for generation ← 1 to number of generations do fitness list
fitness list ← EvaluateFitness(population);
if max(fitness list) ≥ fitness threshold then
Print ”Optimal fitness achieved.”;
return;
end
parents ← Selection(fitness list, population);
children ← Crossover(parents);
Mutation(children);
population ← UpdatePopulation(population, children);
end
Function InitializePopulation(n):
return list of n chromosomes each with randomly initialized parameters;
Function EvaluateFitness(pop):
return list of fitness scores computed using a CNN model based on the chromosome’s parameters;
Function Selection(fitness, pop):
return two selected parents based on roulette wheel selection method;
Function Crossover(parents):
return two offspring produced by single-point crossover from the parents;
Function Mutation(children):
Apply random mutations to the children with a predetermined probability;

## Genetic algorithm for VCNet optimization

**Fig. 4:**
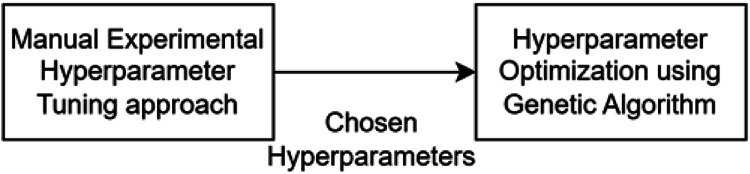
Two-step hyperparameter optimization.

A population of chromosomes, representing the sets of potential hyperparameters, is initialized. The algorithm undergoes generation and assesses the fitness of every chromosome with the CNN model. This would mean that the fitness relates to the performance of the model with the set hyperparameters, something that is always measured by the accuracy or any other metric that might define the performance. If any chromosome reaches a fitness score greater than or equal to some predefined threshold, the algorithm will terminate early by announcing that optimal fitness has been reached. Otherwise, this algorithm selects the parents from the current population by employing a roulette wheel selection in which the selection is made probabilistically based on the relative fitness of each individual. This is followed by the production of offspring using a single-point crossover operator, which merges information from two parent chromosomes. These offspring are then under the process of mutation-introducing random changes to their hyperparameters, which may provide even better solutions. This set of new children then replaces the population, and the cycle repeats until the number of generations has reached its maximum or a fitness threshold has been reached. That can be quite useful when it comes to tuning such complex models, for which hand-picking and trying all the possible hyperparameters would take way too much time. Initially the experiment with adding or removing layers, adjusting layer sizes, or modifying dropout rates is carried to find best hyperparameters manually as depicted in Fig. 4. These hyperparameters are then fed as initial parameters to Genetic algorithm for optimizing the model and hyperparameter as listed in Table 3.

**Table 3:** Parameters for Genetic optimization algorithm.

*Number of Generations*	*Fitness Threshold*	*Number of Populations*	*Filter size*			*Dropout*	*Learning Rate*	*Number of Neurons in Dense layers*
*5*	*90*	*10*	*16,32,64,128*			*0.3,0.4,0.5,0.6*	*0.001,0.003*	[100,150],[125,150],[150,175], [175,250],[250,500]

## Training details

The proposed framework employed custom VGG16 for feature extraction, and therefore the model is trained retaining the VGG16 weights. The batch size of 64 and the categorical cross-entropy loss function are used to train the model till 100 epochs. The genetic algorithm optimizes the hyperparameters. Adam optimizer with 0.003 learning rate is used for training. Retraining of state-of-the-art architectures is also done using the same loss function and optimizer. The Kaggle platform is used to train the model with GPU P100 and 100 mbps Internet connection speed. The system had local 16 GB of Ram and i7 processor.

## Results and discussion

**Fig. 5:**
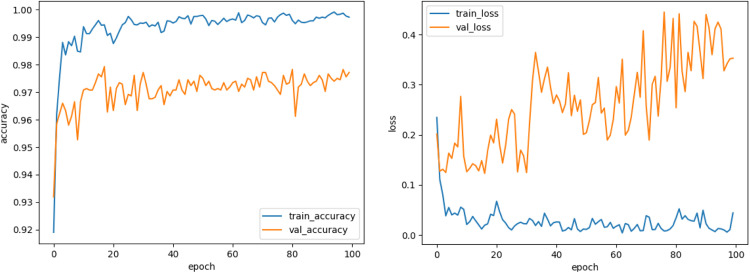
Training accuracy and loss graph.

Observing the training performance for VCNet after 100 epochs, it is seen in Fig. 5 that both the training and validation accuracy curve move in the same direction, hence is evident that there is no overfitting. The training performance has consistently improved with increasing number of epochs and then it is stable. The training loss is 0.044 and validation loss is 0.35 indicating that proposed model is performing accurately. The training performance suggests that model can perform better if trained upto 50 epochs thereby obtaining lesser training time.

The proposed VCNet has predicted the rice diseases with training accuracy of 99.72 % and the testing accuracy of 97.71 %. Table 4 below indicates that performance metrics are having optimal results. The loss value obtained is indicating that proposed model is performing efficiently for rice disease detection. Training time for 100 epochs is 2565 s only.

**Table 4:** Result analysis for proposed VCNet framework with genetic optimization.

***Training*** ***Accuracy (%)***		***Validation*** ***Accuracy (%)***	***Testing Accuracy (%)***	***Training*** ***Loss***	***Validation*** ***Loss***	***Precision***	***Recall***	***F1Score***	***Training*** ***Time(s)***
*99.72*	*97.72*		*97.71*	*0.044*	*0.35*	*0.978*	*0.978*	*0.978*	*2565*

**Fig. 6:**
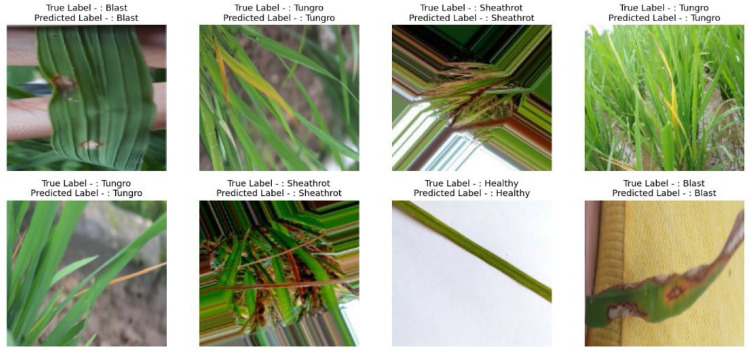
Test predictions for different classes of disease.

The Fig. 6 is the snapshot of predicted results on testing data. It can be seen that the predicted rice crop disease by VCNet model is accurate for all classes of diseases. It is noted that the dataset has different background and variations in different images of different classes of diseases but model has excellent generalization power hence predicting correctly.

### Comparative result analysis with state-of-the-art

**Fig. 7:**
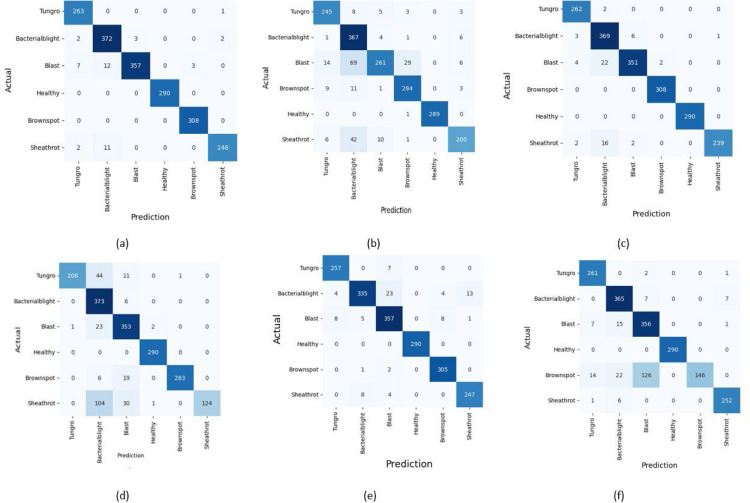
Confusion Matrix (a)Proposed VCNet framework (b)Inceptionv3 (c)VGG16 (d)ResNet50 (e)MobileNetv2 (d)DenseNet121.

It is marked that number of true positive predictions for all classes of rice crop diseases is highest with proposed VCNet model depicted in Fig. 7. Existing models are predicting correctly for some class but poor for some other class whereas proposed VCNet is performing consistently correct for all classes of rice crop diseases. The total number of images for testing were 259 for sheath rot out of which proposed model predicted 246 images correctly whereas Inceptionv3 and VGG16 have a smaller number of correct predictions i.e. 200 and 239. The Resnet50 is performing lowest with only 124 correct predictions while 135 predictions are misclassified. MobileNetv2 and DenseNet121 though achieving similar result as VCNet for sheath rot but they have misclassified other classes of disease. MobileNetv2 has given only 335 out of 379 correct predictions for bacterial blight whereas proposed model has correctly classified 372 for the same. Similarly, DenseNet121 has only 146 out of 308 correct predictions for brown spot disease whereas proposed model has all 308 predictions correct for brown spot.

**Fig. 8:**
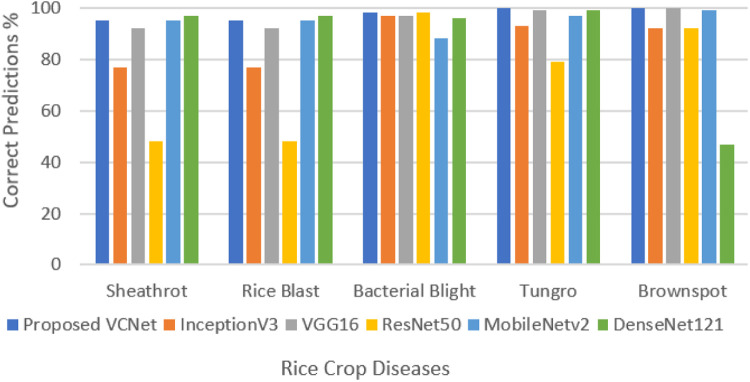
Performance analysis for each class of rice crop disease.

Fig. 8 shows the chart for comparative performance of proposed VCNet with other state of the art models for each rice crop disease. Proposed VCNet is the best performer for multiclass rice disease prediction by predicting highest number of correct predictions for all classes of rice crop diseases as compared to other models who have misclassified one or the other classes of disease in large number. In the figure above it can be seen that Resnet50 is lowest in performance in correctly predicting sheath rot with 48 % only and 79 % for tungro, InceptionV3 is lowest in predicting rice blast with only 69 %, MobileNetv2 is lowest in predicting bacterial blight with 88 % and DenseNet121 is lowest in predicting brown spot with 47 % only. VCNet is performing exceptional for all classes of prediction with 95 % correct predictions for sheath rot, 94 % for rice blast, 98 % for bacterial blight and 100 % for tungro and brown spot.

The proposed VCNet model has 97.71 % percentage of testing accuracy, 99.72 % training accuracy and other performance metrics as shown in Table 5. On comparing with the result of existing models for the trainable parameters required it was seen that the DenseNet121 is having just 98,310 parameters but the time required for training is more than the VCNet. The VCNet is also compared with latest studies for customized models for rice crop disease detection as seen in Table 6 and noted that it is best performer considering greatest accuracy as well as less parameters.

**Table 5:** Comparative result analysis.

*Models*	*Training Accuracy* %	*Testing accuracy* %	*Precision* %	*Recall* %	*F1Score* %	*Parameters*	*Time(secs)*
*Proposed VCNet*	*99.72*	*97.71*	*97.85*	*97.82*	*97.81*	*670,589*	*2565*
*InceptionV3*	*89.74*	*87.59*	*89.43*	*87.93*	*88.01*	*2191,338*	*6520*
*VGG16*	*98.55*	*95.8*	*97.2*	*96*	*97.03*	*329,485*	*3895*
*ResNet50*	*99.56*	*86.8*	*91*	*85*	*86*	*2384,179*	*3400*
*MobileNetV2*	*97.31*	*95.31*	*95.5*	*95.7*	*95.59*	*2449,990*	*3100*
*DenseNet121*	*99.71*	*95.47*	*96.1*	*95.1*	*95.4*	*98,310*	*2940*

**Table 6:** Comparison *with recent methodologies.*

*Paper*	*Model Name*	*Number of Images*	*Parameters*	*Accuracy*
[25], 2024	*DeepRice*	*5932*	*1,49,91,826*	*99.70* %
[26], 2024	*Deep Spectral Generative Adversarial Neural Network*	*1000*		*97* %
[27], 2025	*ConvNeXt*	*3000 images*	*27,800,000*	*94.69* %
	*Proposed VCNet*	*9347*	*670,589*	*99.72* %

The proposed VCNet with custom architecture employing CNN and VGG16, dropout layers and optimal optimized hyperparameters using Genetic algorithm optimization has proved to be efficient design for rice disease classification. The VCNet model is at its best with respect to the performance metrics, time as well as trainable parameter because of its novel optimized framework.

## Conclusion

The rice crop disease detection using deep learning has proved its efficiency in various research studies but the problem of balanced generalization capability of multiclass classification is still challenge. Sheath rot is the most important class of disease under study as it is causing damage in fields every year but still not explored in many studies. It is seen in our study that popular state of the art models like Inceptionv3, ResNet50, DenseNet121 have correctly classified some class of rice crop disease but misclassified other class of rice crop disease at a large scale. Hence to overcome this issue we have proposed novel VCNet, an optimized deep learning framework to correctly predict all classes of rice diseases in multiclass classification efficiently. The proposed framework extracts feature initially with VGG16 and then custom architecture fused with genetic algorithm for optimization of hyperparameters. The optimized framework has better generalization capability as compared to state of the art achieving 95 % for sheath rot, 94 % for rice blast, 98 % for bacterial blight and 100 % for tungro as well as brown spot. The images from sheath rot, rice blast and bacterial blight are collected from rice fields but still model is able to predict efficiently for these classes inspite of variation in dataset images. The training time taken by VCNet is 2565 s which is minimum than the existing state of the art models. The optimized VCNet requiring smaller number of trainable parameters can easily be deployed for applications with limited processing capability. The future research to make it more lightweight would be under consideration.

## Limitations

• The data collection and labelling for real field images is restricted to three rice crop diseases rice blast, bacterial blight and sheath rot due to availability issue.
• The proposed framework is tested for 6 classes of multiclass classification. It can be extended to include more classes of diseases.

## Ethics statements

This work as no human or animal as a subject and no data from social media was needed.

## CRediT authorship contribution statement

**Sanam Salman Kazi:** Conceptualization, Methodology, Data curation, Project administration, Investigation, Validation, Visualization, Writing – original draft, Writing – review & editing. **Bhakti Palkar:** Data curation, Formal analysis, Project administration, Supervision. **Dhirendra Mishra:** Formal analysis, Validation, Supervision.

## Declaration of competing interest

The authors declare that they have no known competing financial interests or personal relationships that could have appeared to influence the work reported in this paper.

## Data Availability

Data will be made available on request.
